# Fine Mapping and Identification of a Novel Phytophthora Root Rot Resistance Locus *RpsZS18* on Chromosome 2 in Soybean

**DOI:** 10.3389/fpls.2018.00044

**Published:** 2018-01-30

**Authors:** Chao Zhong, Suli Sun, Liangliang Yao, Junjie Ding, Canxing Duan, Zhendong Zhu

**Affiliations:** ^1^National Key Facility for Crop Gene Resources and Genetic Improvement, Institute of Crop Sciences, Chinese Academy of Agricultural Sciences, Beijing, China; ^2^Jiamusi Branch of Heilongjiang Academy of Agricultural Sciences, Jiamusi, China

**Keywords:** Phytophthora root rot, *Phytophthora sojae*, resistance gene, *RpsZS18*, soybean

## Abstract

Phytophthora root rot (PRR) caused by *Phytophthora sojae* is a major soybean disease that causes severe economic losses worldwide. Using soybean cultivars carrying a *Rps* resistance gene is the most effective strategy for controlling this disease. We previously detected a novel Phytophthora resistance gene, *RpsZS18*, on chromosome 2 of the soybean cultivar Zaoshu18. The aim of the present study was to identify and finely map *RpsZS18*. We used 232 F_2:3_ families generated from a cross between Zaoshu18 (resistant) and Williams (susceptible) as the mapping population. Simple sequence repeat (SSR) markers distributed on chromosome 2 were used to map *RpsZS18*. First, 12 SSR markers linked with *RpsZS18* were identified by linkage analyses, including two newly developed SSR markers, ZCSSR33 and ZCSSR46, that flanked the gene at distances of 0.9 and 0.5 cM, respectively. Second, PCR-based InDel markers were developed based on sequence differences between the two parents and used to further narrow down the mapping region of *RpsZS18* to 71.3 kb. Third, haplotype analyses were carried out in the *RpsZS18* region using 14 soybean genotypes with whole-genome resequencing. We detected six genes with unique haplotype sequences in Zaoshu18. Finally, quantitative real-time PCR assays of the six genes revealed an EF-hand calcium-binding domain containing protein encoding gene (*Glyma.02g245700*), a pfkB carbohydrate kinase encoding gene (*Glyma.02g245800*), and a gene with no functional annotation (*Glyma.02g246300*), are putative candidate PRR resistance genes. This study provides useful information for breeding *P. sojae*-resistant soybean cultivars.

## Introduction

Phytophthora root rot (PRR), caused by the soil-borne oomycete *Phytophthora sojae* (Kanfman and Gerdemann), is a devastating disease in most soybean-growing regions. *P. sojae* can infect soybean plants at all growth stages under saturated soil conditions (Schmitthenner, 1985). PRR usually causes yield losses of 30–50% under favorable conditions, but during severe epiphytotic outbreaks infections can result in 100% yield loss (Li and Ma, 1999; Zhang et al., 2010). In China, PRR was first detected in Heilongjiang Province in 1989, and now has spread to most soybean-producing areas (Zhu et al., 2003; Chen and Wang, 2017). The use of resistant cultivars is the most economical and environmentally safe method to control this disease.

Two types of resistance to *P. sojae* have been identified in soybean, namely complete and partial resistance (Sugimoto et al., 2012). Complete resistance is race-specific, and is the result of a single dominant resistance gene (*Rps*) that confers immunity or near immunity. Partial resistance is controlled by major and minor genes and limits pathogen colonization and spread (Tooley and Grau, 1984; Dorrance et al., 2003b, 2004; Sugimoto et al., 2012). Currently, the best method to control PRR involves growing soybean cultivars with complete resistance because partial resistance to *P. sojae* is ineffective under conditions of high disease pressure (Schmitthenner, 1999; Dorrance et al., 2003b). The first *Rps* gene (*Rps1a*) was identified in soybean in the 1950s (Bernard et al., 1957), and since then, more than 20 *Rps* genes have been identified and mapped to nine chromosomes, namely chromosomes 2, 3, 7, 10, 13, 14, and 16–19 (Sugimoto et al., 2012; Lin et al., 2013; Zhang et al., 2013a,b; Sun et al., 2014; Ping et al., 2015; Li et al., 2016; Cheng et al., 2017; Li Y. et al., 2017; Niu et al., 2017; Qin et al., 2017; Sahoo et al., 2017; Zhong et al., 2017). Some soybean cultivars and lines carrying *Rps* genes, including *Rps1a, Rps1c*, and *Rps1k*, have been used widely in commercial breeding programs to reduce yield losses caused by PRR (Dorrance and Schmitthenner, 2000; Sugimoto et al., 2012). Among the identified *Rps* genes, only *Rps1k* has been cloned and characterized. *Rps1k* was mapped in a 125-kb region through the construction of high resolution genetic and physical maps (Kasuga et al., 1997). A gene family encoding a nucleotide binding site-leucine-rich repeat (NBS-LRR) structure has been isolated from the region of *Rps1k* by screening bacterial artificial chromosome (BAC) libraries, and two NBS-LRR-encoding genes (*Rps1k-1* and *Rps1k-2*) were cloned (Gao et al., 2005; Gao and Bhattacharyya, 2008). However, the exact physical location of *Rps1k* remains unknown in the soybean Williams 82 (carries *Rps1k*) reference genome (Lin et al., 2013; Sun et al., 2014; Li et al., 2016), and this has also hindered studies of the mechanism of soybean resistance to *P. sojae*.

Previous studies have described a typical gene-for-gene interaction between soybean and *P. sojae*. More than 200 *P. sojae* physiological races or pathotypes have been identified since 1955 (Schmitthenner et al., 1994; Ryley et al., 1998; Dorrance et al., 2003a; Sugimoto et al., 2012; Stewart et al., 2014, 2015; Xue et al., 2015). In China, there is considerable diversity in the virulence of isolates from different regions, especially those from the Huanghe-Huaihe and Yangtze basins (Zhu et al., 2003). Among the known *Rps* genes, only *Rps1c, Rps1k*, and *RpsYD25* can control PRR in the major soybean-producing regions of China (Zhu et al., 2003; Fan et al., 2009; Zhang et al., 2010; Li Y. et al., 2017). However, no *Rps* gene that confers resistance to all *P. sojae* races or pathotypes has been found in China to date (Zhu et al., 2003; Cui et al., 2010; Zhang et al., 2010, 2014). Therefore, it is imperative that novel *Rps* genes effective against new *P. sojae* races or pathotypes are identified and incorporated into commercial cultivars. Developing markers closely linked to *Rps* genes is an efficient way to use marker-assisted selection to breed cultivars with stable and durable resistance to *P. sojae*.

The sequencing and assembly of the soybean Williams 82 reference genome and the refinement of gene annotations have facilitated the development of more molecular markers and the discovery of candidate *Rps* genes (Grant et al., 2009; Schmutz et al., 2010; Zhang et al., 2013a,b; Li et al., 2016; Song et al., 2016; Cheng et al., 2017; Li Y. et al., 2017). High-density simple sequence repeat (SSR) markers were developed based on the Williams 82 reference genome by Song et al. (2010), and since then, many *Rps* genes has been identified and finely mapped including *RpsJS, Rps11*, and *RpsHN* (Lin et al., 2013; Sun et al., 2014; Ping et al., 2015; Niu et al., 2017). Based on the published Williams 82 genomic sequence information, new SSR markers have been developed to build higher-resolution linkage maps, and novel *Rps* genes like *RpsYD29* and *Rps10* have been identified (Zhang et al., 2013a,b). In recent years, the reduced costs of sequencing and application of bioinformatic analysis based on next-generation sequencing, have allowed single nucleotide polymorphism (SNP) and insertion/deletion (InDel) markers to be identified and applied to the mapping population (Li et al., 2016; Li Y. et al., 2017).

Zaoshu18 is an elite soybean cultivar, which was grown in the Huanghuaihai region of China during the 1980s. In previous studies, we found that Zaoshu18 exhibited excellent resistance to PRR (Zhu et al., 2006; Chen et al., 2008; Zhang et al., 2014). Our initial study revealed that Zaoshu18 resistance to *P. sojae* was controlled by a single dominant gene, *RpsZS18*, which preliminarily mapped on chromosome 2 (Yao et al., 2010). However, because of the lack of polymorphic markers and genomic sequence information, only a few SSR markers with distant genetic distances were found, so an accurate genetic linkage map could not be constructed. Further, in the wide mapping interval of the previous map (Yao et al., 2010), none of the typical NBS-LRR-encoding disease resistance genes were found. Therefore, a novel *Rps* gene structure with a different *P. sojae* resistance mechanism may exist on soybean chromosome 2. The objectives of this study were (1) to develop markers closely linked to *RpsZS18* conferring PRR resistance, (2) to finely map *RpsZS18* on chromosome 2, and (3) to analyze *RpsZS18* candidate gene(s) based on whole-genome resequencing of 10 soybean genotypes, including two contrasting parents.

## Materials and methods

### Plant materials and *P. sojae* isolates

Phenotyping was performed for Zaoshu18 and 20 differential cultivars containing a single *Rps* gene. Four cultivars, Williams, Zhonghuang13, Zhonghuang47, and Jikedou2, were used as susceptible controls. The F_1_ soybean seeds used in this study were produced from a cross between the susceptible cultivar Williams and the resistant cultivar Zaoshu18, which carries the PRR resistance gene *RpsZS18*. The F_1_ plants were self-pollinated to produce an F_2_ population consisting of 232 individuals. Each F_2_ plant was self-crossed and threshed individually to generate seeds for 232 families, which were used for phenotypic and genotypic evaluations.

The phenotype test was conducted using a total of 14 *P. sojae* isolates with different pathotypes. All the different isolates were cultured on V_8_ juice agar medium. The *P. sojae* isolates PsFJ2, PsFJ3, Ps41-1, and PsUSAR2 were used for phenotypic evaluations of the parents and the F_2:3_ population.

### Evaluation of PRR in the F_2:3_ population

We planted 15–20 seeds of each cultivar in 10-cm diameter paper cups, with 14 pots for each cultivar. After 12 days, the seedlings were inoculated with 14 *P. sojae* isolates, one for each pot, using a modified hypocotyl-inoculation technique described by Zhang et al. (2013a). Briefly, a 5-mm incision was made in the hypocotyls, into which the slurry of *P. sojae* inoculum, made from a 7-day-old V_8_ juice agar culture, was inserted. Inoculated seedlings were placed in a mist chamber at 24°C with 100% relative humidity for 2 days. The plants were then moved to a greenhouse and grown at 23–26°C. After a 4-day incubation, the numbers of dead seedlings were recorded. Cultivars were considered resistant if all the seedlings were alive with no expanded lesions. Cultivars were considered susceptible if all the seedlings were dead.

For phenotypic evaluation of the F_2:3_ population, we planted 25–30 seeds from each of the 232 families in 10-cm diameter paper pots, with two pots per family. The parents were planted separately as controls. After a 4-day incubation, the numbers of dead seedlings were recorded. Families with 0–20, 80–100, and 21–79% dead seedlings were considered homozygous resistant, homozygous susceptible, and segregating, respectively (Gordon et al., 2006; Lin et al., 2013; Zhang et al., 2013a,b; Ping et al., 2015). All cultivars, parents, and families were tested twice. If the results were inconsistent in the two tests, a third test was conducted to confirm the accuracy of the phenotype.

### SSR marker development and analyses

Equal amounts of leaf tissue from 25 to 30 seedlings of each family and parental cultivars were collected and pooled together before inoculation. Each of 25–30 individual plants from one family was ensured to be harvested leaf tissue. Genomic DNA of each family was extracted using a Plant Genomic DNA Kit (Tiangen, Beijing, China).

To map the *RpsZS18* gene, 28 published SSR markers from SoyBase (https://www.soybase.org/) and 154 new SSR markers were used to screen for polymorphisms between parents according to the rough mapping interval of Yao et al. (2010). The 154 new SSR markers were designed based on the chromosome 2 sequence of Williams82 (*Glycine max* V2.0) in SoyBase (https://www.soybase.org). Novel SSR motifs were screened using the SSR Hunter 1.3 program, and SSR primers were designed with Primer Premier 5.0. All SSR primer pairs were synthesized by Sangon Biotech (Beijing, China). Subsequently, polymorphic SSR markers were used to analyze the genotypes of the 232 F_2:3_ families.

Each polymerase chain reaction (PCR) was completed in a 10-μl sample consisting of 30 ng genomic DNA, 5 μl 2 × Taq PCR MasterMix (Tiangen), and each primer (0.2 μM). The PCRs were conducted in a thermal cycler (Biometra, Gottingen, Germany) using the following program: 95°C for 3 min; 35 cycles of 95°C for 45 s, 52–58°C for 45 s, and 72°C for 45 s; 72°C for 7 min. Samples were cooled to 10°C. The PCR products were mixed with 2 μl 6 × loading buffer (0.25% bromophenol blue, 25% xylene cyanol FF, and 40% sugar) and separated in 8% non-denaturing polyacrylamide gels.

### Data analyses and linkage map construction

The segregation of phenotypes and molecular markers in the F_2:3_ population was evaluated for goodness-of-fit to Mendelian segregation ratios using the Chi-squared test. Linkage analyses were completed using MAPMAKER/EXP version 3.0 (Lincoln et al., 1993). Genetic distances were calculated according to the Kosambi mapping function (Kosambi, 1943). Linkage groups were determined using a log-likelihood threshold of 3.0. The genetic linkage map of molecular markers linked to *RpsZS18* was prepared using MapDraw (Liu and Meng, 2003).

### Whole-genome resequencing of soybean cultivars

We performed whole-genome resequencing (WGRS) of the two parent cultivars, Zaoshu18 and Williams, and 12 different soybean cultivars with or without a known *Rps* gene. Genomic DNA was extracted from the 14 cultivars to construct Illumina libraries, which were sequenced by Biomarker Technologies and Annoroad Gene Technology (Beijing, China). The raw 150 paired-end reads data were filtered to produce clean reads as follows: (1) removal of reads with adapters, reads containing more than 15% bases with a Phred quality score <19, and reads with more than 5% undetermined bases; (2) alignment of filtered clean reads to the Williams82 reference genome (*Glycine max* V2.0) using the genome-alignment-software BWA; and (3) calculation of mapping rate, 5× genome coverage, and mean depth (Li and Durbin, 2009).

SNPs and InDels were detected using the SNP analyses software GATK (McKenna et al., 2010). ANNOVAR software and the existing genomic annotation file (gff/gtf) were used to annotate the SNPs (Wang et al., 2010). DELLY (Rausch et al., 2012) was used for the analysis of chromosome structure variation (SV). All potential SV sites were detected and filtered for genomewide testing. By comparing the reads with the reference genome, we identified all variants, including SNPs, InDels, and SVs, among the samples.

### Fine mapping with InDels

Based on the genetic linkage map constructed with SSR markers, the physical region of *RpsZS18* was determined according to the reference genome sequence of Williams 82 (*Glycine max* V2.0). InDels in the *RpsZS18* mapping region were identified between the parental cultivars Williams and Zaoshu18. PCR-based InDel markers were developed in the mapping region of *RpsZS18* using Primer-BLAST (https://www.ncbi.nlm.nih.gov/tools/primer-blast/). F_2:3_ families and parental cultivars were genotyped based on the InDel markers. PCR reactions were conducted as described above for the SSR markers. New recombination sites in the F_2:3_ population were discovered using the InDel markers, and the candidate region for *RpsZS18* was shortened.

### Resistance-related variation identification and candidate gene prediction

To identify the *RpsZS18* disease resistance candidate gene, the Homozygous SNPs, InDels and high confident SVs in the physical mapping interval of *RpsZS18* corresponding to the 14 different soybean cultivars with whole-genome sequences were selected for haplotype analyses. The identified variants were used to develop reference-based assembly of corresponding *RpsZS18* allelic regions among the 14 sequenced soybean cultivars by substituting the bases with confident variant calls in the reference genome (Singh et al., 2016a,b). Haplotype sequences of the 15 soybean cultivars were compared to each other to identify specific variations of Zaoshu18. Website GeneScan (http://genes.mit.edu/GENSCAN.html) was used to predict whether these variations constitute additional gene models.

### Expression analyses of candidate genes

Zaoshu18 and Williams seedlings were inoculated with the isolate PsUSAR2 when the first pair of true leaves had expanded completely. We collected 1-cm samples from above and below the treated hypocotyl tissues at 0, 6, 12, 24, 48, and 72 h post inoculation, and stored them at −80°C. For the analysis of tissue-specific transcript abundance, the roots, stems and leaves were also collected at 0 h post inoculation. Allele expression patterns were tested for the 14 sequenced soybean cultivars and Williams at 24 h post inoculation. Three individuals were mixed at each time point for total RNA extraction. Total RNA was isolated using an RNAprep Pure Plant Kit (Tiangen Biotech, Beijing, China), and cDNA was synthesized using a PrimeScript™RT Reagent Kit (TaKaRa, Japan). Specific primers for the quantitative real-time PCR (qRT-PCR) were designed according to the conserved coding region between the haplotype sequences using Primer-BLAST (https://www.ncbi.nlm.nih.gov/tools/primer-blast/). The housekeeping gene *Actin11* was used as an internal control. Gene expression was determined by qRT-PCR using SYBR® Premix Ex TaqII (TliRNaseH Plus) (TaKaRa, Japan) on an ABI 7500 platform (Applied Biosystems, USA). The 2^−ΔΔCT^ method was used to calculate the relative expression levels of candidate genes (Livak and Schmittgen, 2001). Three biological replicates with their respective three technical replicates were conducted for each sample.

## Results

### Phenotypic evaluation

**Figure 1:**
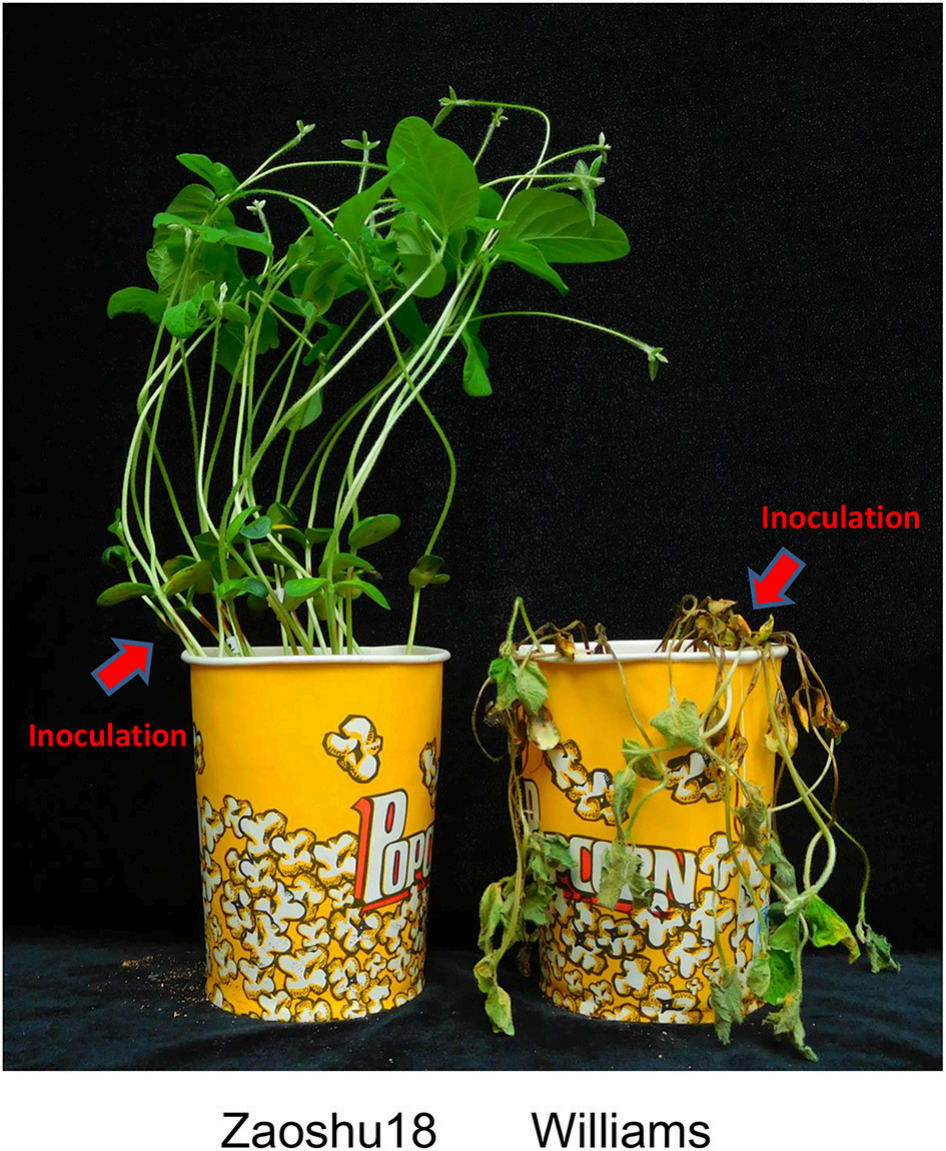
Phytophthora resistance phenotype of the soybean cultivars Zaoshu18 and Williams were tested with isolate PsUSAR2 using the hypocotyl-inoculation technique for 6 days. All the Williams soybean plants (susceptible parent) were fallen and dead, whereas all the Zaoshu18 plants (resistant parent) were alive and exhibited no disease symptoms. The red arrow represents the inoculation site, in which the lesion invades and spreads along the hypocotyl in the susceptible cultivar Williams, whereas the resistant cultivar Zaoshu18 shows an immune response with no spread of the lesion.

All the plants of the four susceptible controls, Williams, Zhonghuang13, Zhonghuang47, and Jikedou2, were dead after being inoculated individually with each of the 14 *P. sojae* isolates. The other 20 resistant cultivars carrying a single known *Rps* gene were resistant to 3–12 isolates. Zaoshu18 was resistant to 10 of the 14 *P. sojae* isolates (Table 1). A total of 17 reaction types among the 25 soybean cultivars were formed in response to the 14 *P. sojae* isolates, and Zaoshu18 showed a distinct reaction type. This suggested that Zaoshu18 contained a novel *Rps* gene or genes combination.To test the phenotypes of the F_2:3_ population, Four *P. sojae* isolates, PsFJ2, PsFJ3, PsUSAR2, and Ps41-1, were selected for inoculation. Six days after being inoculated with the four isolates, all the Williams soybean plants (susceptible parent) were dead, whereas all the Zaoshu18 plants (resistant parent) were alive and exhibited no disease symptoms (Figure 1). Among the 232 F_2:3_ families of the mapping population, the ratio of homozygous resistant to segregating to homozygous susceptible for four isolates PsFJ2, PsFJ3, PsUSAR2, and Ps41-1 was 54:112:66, which fitted the expected 1:2:1 ratio (Table 2). Thus, resistance to the four isolates PsFJ2, PsFJ3, PsUSAR2, and Ps41-1 in Zaoshu18 was confirmed to be controlled by a single dominant gene.

**Table 1:** Phenotypic test of 25 differential cultivars to 14 *P. sojae* isolates.

**Cultivar/lines (*Rps* gene)**	***Phytophthora sojae isolates***													
	**PsRace1**	**PsRace3**	**PsRace4**	**PsRace5**	**PsUSAR2**	**Ps41-1**	**PsAH4**	**PsMC1**	**PsNKI**	**PsFJ2**	**PsFJ3**	**PsJS2**	**Ps6497**	**Ps7063**
Harlon (*Rps1a*)	S	S	S	S	R	S	R	S	R	S	S	S	R	S
Harosoy13XX (*Rps1b*)	R	R	R	R	S	S	S	S	S	S	S	S	S	R
Williams79 (*Rps1c*)	R	R	R	R	R	S	R	R	R	R	R	S	R	R
PI103091 (*Rps1d*)	R	S	S	R	R	S	S	S	S	S	S	S	S	S
Williams82 (*Rps1k*)	R	R	R	R	R	R	S	S	R	R	S	S	R	R
L76-988 (*Rps2*)	R	R	R	R	S	S	S	S	S	S	S	S	S	S
L83-570 (*Rps3a*)	R	R	R	R	R	S	S	S	S	S	S	S	R	S
PRX146-36 (*Rps3b*)	R	R	S	R	R	S	S	S	S	S	S	S	R	R
PRX145-48 (*Rps3c*)	R	R	R	R	S	S	S	S	S	S	S	S	S	R
L85-2352 (*Rps4*)	R	R	R	R	R	S	S	S	S	S	S	S	R	S
L85-3059 (*Rps5*)	R	R	R	R	S	S	S	S	S	S	S	S	R	S
Harosoy62XX (*Rps6*)	R	R	R	R	R	S	R	S	S	S	S	S	R	S
Harosoy (*Rps7*)	R	R	R	S	R	S	S	S	S	S	S	S	S	S
PI399073 (*Rps8*)	R	R	R	R	R	S	R	S	S	S	S	S	R	S
Youbian30 (*RpsYB30*)	R	R	S	R	R	R	S	S	R	R	S	S	S	S
Yudou25 (*RpsYD25*)	R	R	R	R	R	R	S	R	R	S	R	S	R	R
Yudou29 (*RpsYD29*)	R	R	R	R	R	R	S	R	R	R	R	S	R	R
Ludou4 (*Rps9*)	R	R	R	R	R	R	R	S	R	R	S	R	R	R
Qichadou 1 (*RpsQ*)	R	R	R	R	R	R	R	S	R	R	S	R	R	R
Wandou15 (*Rps10*)	R	R	R	R	R	R	R	R	R	S	R	S	R	S
Zaoshu18 (*RpsZS18*)	R	R	R	R	R	R	S	S	R	R	R	S	R	S
Zhonghuang13	S	S	S	S	S	S	S	S	S	S	S	S	S	S
Zhonghuang 47	S	S	S	S	S	S	S	S	S	S	S	S	S	S
Williams (*rps*)	S	S	S	S	S	S	S	S	S	S	S	S	S	S
Jikedou 2	S	S	S	S	S	S	S	S	S	S	S	S	S	S

**Table 2:** Genetic segregation in response to *Phytophthora sojae* isolates in 232 F_2:3_ families derived from a cross between soybean cultivars Zaoshu18 and Williams.

**Parent and the cross**	**Generation**	**Amount**	**Observed number**			**Chi squared tests**		
			**R^a^**	**Rs**	**S**	**Expected ratio**	**χ^2^**	***P***
Zaoshu18	P1	25	25	–	–			
Williams	P2	25	–	–	25			
Williams × Zaoshu18	F3	232	54	112	66	1:2:1	1.52	0.47

*R^a^, homozygous resistant; Rs, segregating; S, homozygous susceptible*.

### Linkage analyses and genetic mapping

**Figure 2:**
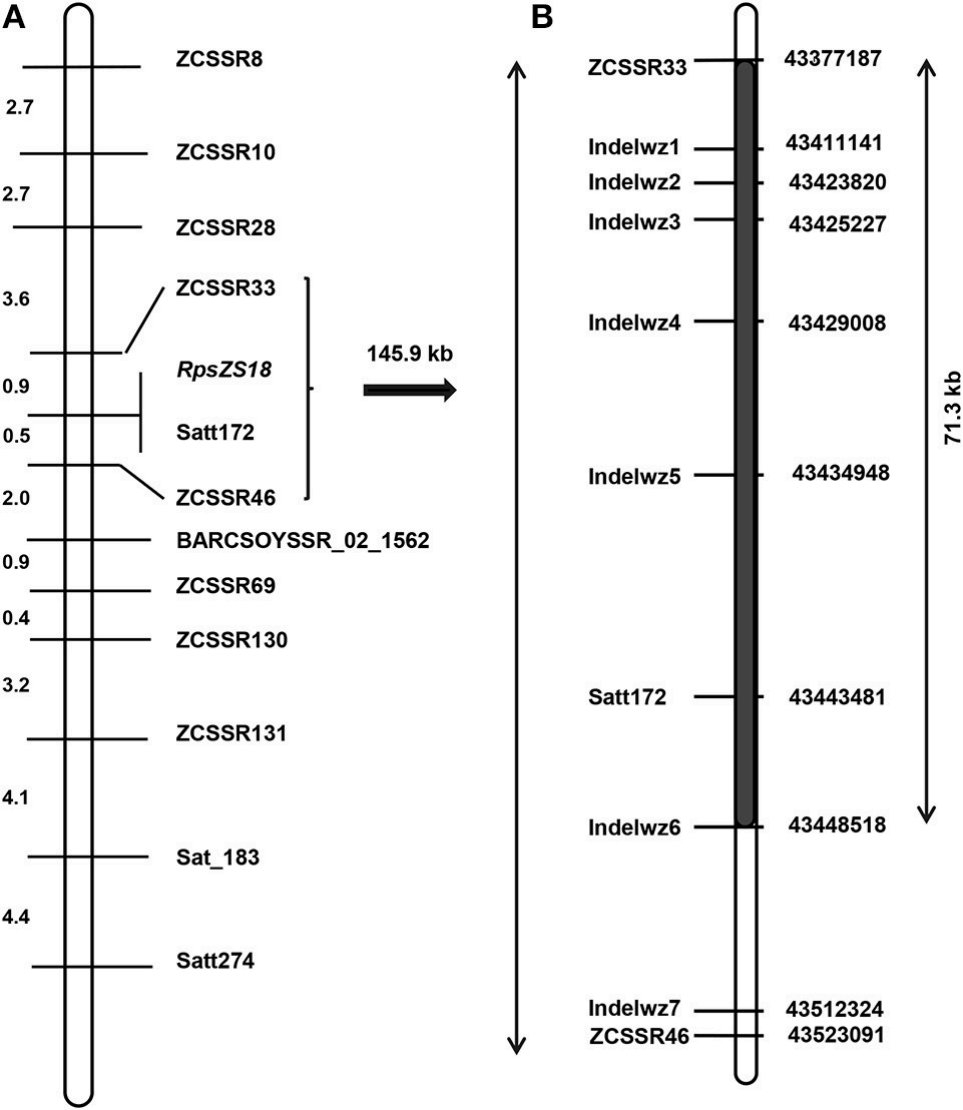
Genetic and physical map of the *RpsZS18* region. **(A)** Linkage map of 12 polymorphic SSR markers linked to *RpsZS18* on soybean chromosome 2. Genetic distances, in cM, are shown on the left, and the locations of the markers and *RpsZS18* are indicated on the right. **(B)** The physical map of the *RpsZS18* which was constructed by genotyping of InDel markers between ZCSSR33 and ZCSSR46. The black bar represents the 71.3-kb region in which no recombination events happened in the F_2:3_ population. The locations of InDel markers are on the left, and the physical position of the chromosome 2 are provided on the right.

We selected 28 published SSR markers distributed on chromosome 2 to genotype the Zaoshu18 and Williams. Four of the 28 SSR markers, namely Satt172, Sat_183, Satt274, and BARCSOYS_02_1562, were polymorphic (Table 3). Subsequently, 154 SSR markers were developed in the target region, and eight of them (ZCSSR8, ZCSSR10, ZCSSR28, ZCSSR33, ZCSSR46, ZCSSR69, ZCSSR130, and ZCSSR131) were polymorphic between the parents, Zaoshu18 and Williams. A total of 12 polymorphic SSR markers were used to genotype the F_2:3_ population for linkage analyses (Figure 2A). All 12 markers were linked to *RpsZS18*; ZCSSR33, and ZCSSR46 flanked *RpsZS18* at genetic distances of 0.9 and 0.5 cM, and Satt172 co-segregated with *RpsZS18* (Figure 2A).

**Table 3:** Primers for the PCR-based SSR markers linked to *RpsZS18* on soybean chromosome 2.

**Markers**	**Primer sequence (5′−3′)**	**Position**		**Motif**	**Fragment**	**Expected**	
		**Start**	**End**		**Size (bp)**	***χ^2^***	***P***
ZCSSR8	F: CGAAGGAAGCCAAAGGAT	43310747	43310946	(GTG)_7_	200	0.59	0.75
	R: CCGCCTCACTGGCTTATT						
ZCSSR10	F:AGGCGGGTGGTAGTTGTA	43312700	43312949	(AT)_25_	250	0.94	0.63
	R:TGGTAAATAGTAAAAGCA						
ZCSSR28	F:GCAGAGAGAGACAAAGGGG	43366813	43367023	(AAG)_5_	211	1.18	0.56
	R:CCGTTTGCCATTCGTTGT						
ZCSSR33	F:TAGTTGATAGCACCTGGGGACA	43377187	43377343	(TA)_5_	157	0.83	0.66
	R:TTCTCAGTCTCAAATGCC						
Satt172	F:AGCCTCCGGTATCACAG	43443481	43443700	(AAT)_19_	220	1.52	0.47
	R:CCTCCTTTCTCCCATTTT						
ZCSSR46	F:AAAGGGAGAAGCAAGTAAT	43522898	43523091	(GT)_16_	194	1.49	0.48
	R:TCGCAAACAGTAAACACG						
BARCSOY-SSR_02_1562	F:CCCCCAAAGCGAAAAATAA	43656010	43656307	(TA)_16_	298	2.32	0.32
	R:CGATTCTATAATGGCGCTGTC						
ZCSSR69	F:TTTTGGTCCATCAAGGGTA	43670051	43670293	(TC)_6_	243	3.35	0.19
	R:GCAAGCCATAGATAAGAA						
ZCSSR130	F:TCCCCACAGTAACATAACA	44017408	44017605	(AC)_10_	198	3.36	0.19
	R:TTAGGCGTTTACTTCAGG						
ZCSSR131	F:GAGTAAGATTGGGACAGA	44018687	44018852	(AC)_24_	166	3.17	0.21
	R:CTTTCTCATAAGCCATCT						
Sat_183	F:GCGTCCAGCCTGACCATTTTA	44317044	44317314	(AT)_28_	271	2.46	0.30
	R:GCGTTCCAATGTCTGATTATTT						
Satt274	F:GCGGGGTCAATTAGTTTTCGTCAGTT	45267040	45267222	(TAT)_18_	183	1.43	0.49
	R:GCGCACGGTATATAATCGAACCTAT						

### InDel marker analyses in the *RpsZS18* mapping region

*RpsZS18* was mapped between SSR markers ZCSSR33 and ZCSSR46, which covered ~145.9 kb in the Williams82 reference genome sequence (*Glycine max* V2.0) (Table 3). According to the Glyma 2.0 annotations, there are 19 genes in the target region (Table S1). To narrow the mapped region of *RpsZS18* and identify the candidate genes controlling PRR resistance, PCR-based InDel makers were developed in the *RpsZS18* mapping region based on the InDels identified by WGRS between the two parents, Williams and Zaoshu18. Seven InDel markers could clearly distinguish polymorphisms among the 232 F_2:3_ families using polyacrylamide gels (Table S2). Among the seven InDel markers, five (Indelwz1, Indelwz2, Indelwz3, Indelwz4, and Indelwz5) co-segregated with *RpsZS18* and no recombination events were found in the 232 F_2:3_ families (Figure 2B). SSR marker ZCSSR33 and InDel maker Indelwz6 identified two and one recombinants in the mapping population, respectively, and identifed a 71.3-kb physical interval of *RpsZS18*.

### Haplotype analyses of *RpsZS18* candidate genes

**Figure 3:**
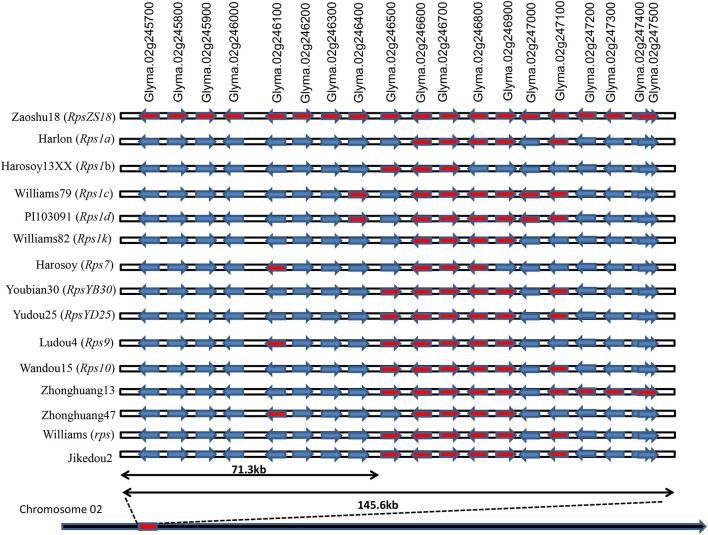
Haplotype sequences comparing between Zaoshu18 and other soybean genotypes in the corresponding *RpsZS18* mapping interval. Gene models marked red were the genes which shared the same haplotype sequences with Zaoshu18, while ones marked blue were the genes with the different haplotype sequences compared to Zaoshu18.

To identify the candidate genes conferring the resistance to PRR in Zaoshu18, based on homozygous SNPs, InDels, and high confident SVs identified by the WGRS, the 145.9-kb *RpsZS18* allelic regions of the 14 sequenced cultivars were constructed by reference-based assembly (Table S3, Supplementary Materials S1). Each soybean cultivar generated 20–25 Gb of clean base data with 70–145 million paired-end 150-bp reads, which resulted in an average depth of 17–22 × genome coverage. Each of these samples had a 5× genome coverage rate higher than 95% (Table 4). In this interval, no SV was found among the 14 sequenced cultivars. Totally, 447 SNPs and 141 InDels were identified in the candidate region of *RpsZS18* among the 15 soybean genotypes. Haplotype sequences of the corresponding *RpsZS18* region were constructed for the 14 sequenced soybean cultivars. As analyzed the haplotype sequences among the 15 soybean genotypes, no additional, extended or truncated genes were identified. Haplotype sequences of the 14 soybean genotypes and Williams82 were aligned to identify the haplotype specific candidate genes for *RpsZS18*. The haplotype sequence of Zaoshu18 was compared with the other 13 soybean genotypes and the reference genotypes Williams82, among the 19 annotated gene models, 13 gene sequences with flanking 1-kb region of Zaoshu18 were consistent with the at least one haplotype sequences of the genotypes, while the other six genes are unique haplotype sequences of Zaoshu18, and all of six genes were in the 71.3-kb interval of *RpsZS18* flanked by ZCSSR33 and Indelwz6 (Figure 3). The six gene models containing Zaoshu18-specific sequences were predicted as candidate genes of *RpsZS18*. Among them, *Glyma.02g245700* and *Glyma.02g245900* encodes elongation factor (EF)-hand calcium-binding domain containing protein; *Glyma.02g245800* encodes a sugar kinase; *Glyma.02g246000* encodes a serine/threonine protein kinase with a leucine-rich repeat (STK-LRR) structure; *Glyma.02g246200* and *Glyma.02g246300* have no annotations so far.

**Table 4:** Whole-genome resequencing of 14 soybean cultivars by Illumina sequencing.

**Genotype sample**	**Clean base (Gb)**	**Number of clean reads**	**Mapping rate (%)**	**5× Genome Coverage Rate (%)**	**Mean Depth (×)**
Zaoshu 18 (*RpsZS18*)	20.05	69954259	99.22	93.53	17
Williams(*rps*)	24.51	85152854	99.28	96.59	21
Zhonghuang13	25.07	99506075	95.42	95.42	22
Zhonghuang47	24.33	96549570	99.16	96.73	21
Jikedou2	26.7	106902333	99.34	96.56	20
Harlon (*Rps1a*)	21.6	144223638	98.17	97.34	22
Harosoy13XX (*Rps1b*)	21.8	145408800	97.65	95.22	22
Williams79 (*Rps1c*)	21.4	142931318	97.08	95.48	22
PI103091 (*Rps1d*)	21.5	143049326	97.07	96.21	22
Harosoy (*Rps7*)	20.3	135309736	97.66	95.22	21
Ludou4 (*Rps9*)	20.95	139679198	96.82	93.07	21
Yudou25 (*RpsYD25*)	21.15	83937297	99.08	95.08	18
Youbian30 (*RpsYB30*)	20.99	74149056	98.9	95.84	19
Wandou15 (*Rps10*)	22.77	79452816	99.04	95.91	20

### Expression analyses of candidate genes

**Figure 4:**
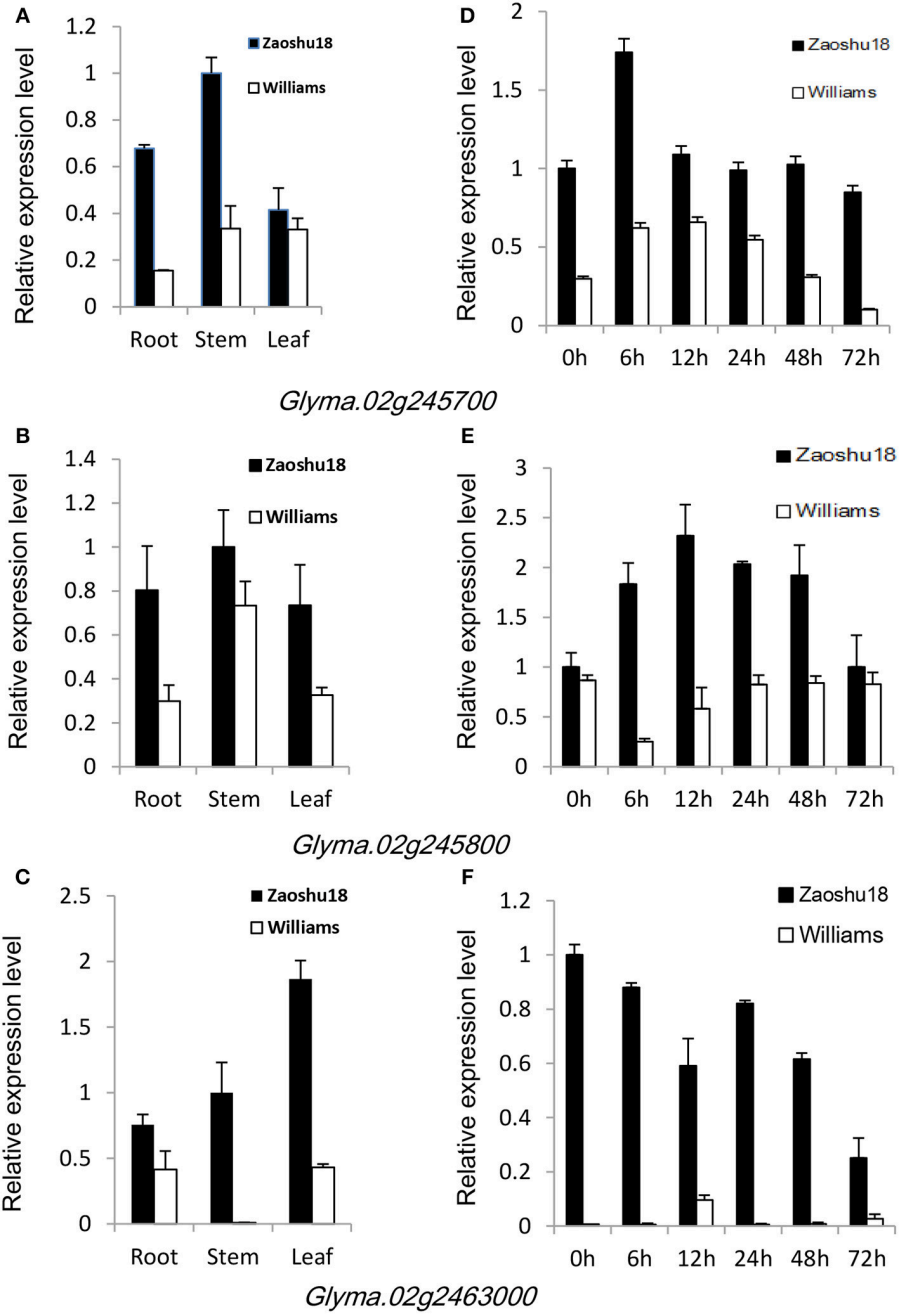
Expression patterns of *Glyma.02g245700, Glyma.02g245800*, and *Glyma.02g246300* in Zaoshu18 and Williams. Each sample was mixed with three individuals at each time point. Three biological replicates with their respective three technical replicates were performed. Bars indicated standard error of the mean. **(A)** Expression patterns of *Glyma.02g245700* in various tissues of Zaoshu18 and Williams. Roots, stems, and leaves were harvested from 12-day-old seedlings without inoculation. **(B)** Relative expression levels of *Glyma.02g245700* in Zaoshu18 and Williams. Infected samples were collected at 0, 6, 12, 24, 48, and 72 h post-inoculation with *P. sojae* isolate PsUSAR2. **(C)** Expression patterns of *Glyma.02g245800* in various tissues of Zaoshu18 and Williams. **(D)** Relative expression levels of *Glyma.02g245800* in Zaoshu18 and Williams. **(E)** Expression patterns of *Glyma.02g246300* in various tissues of Zaoshu18 and Williams. **(F)** Relative expression levels of *Glyma.02g246300* in Zaoshu18 and Williams.

**Figure 5:**
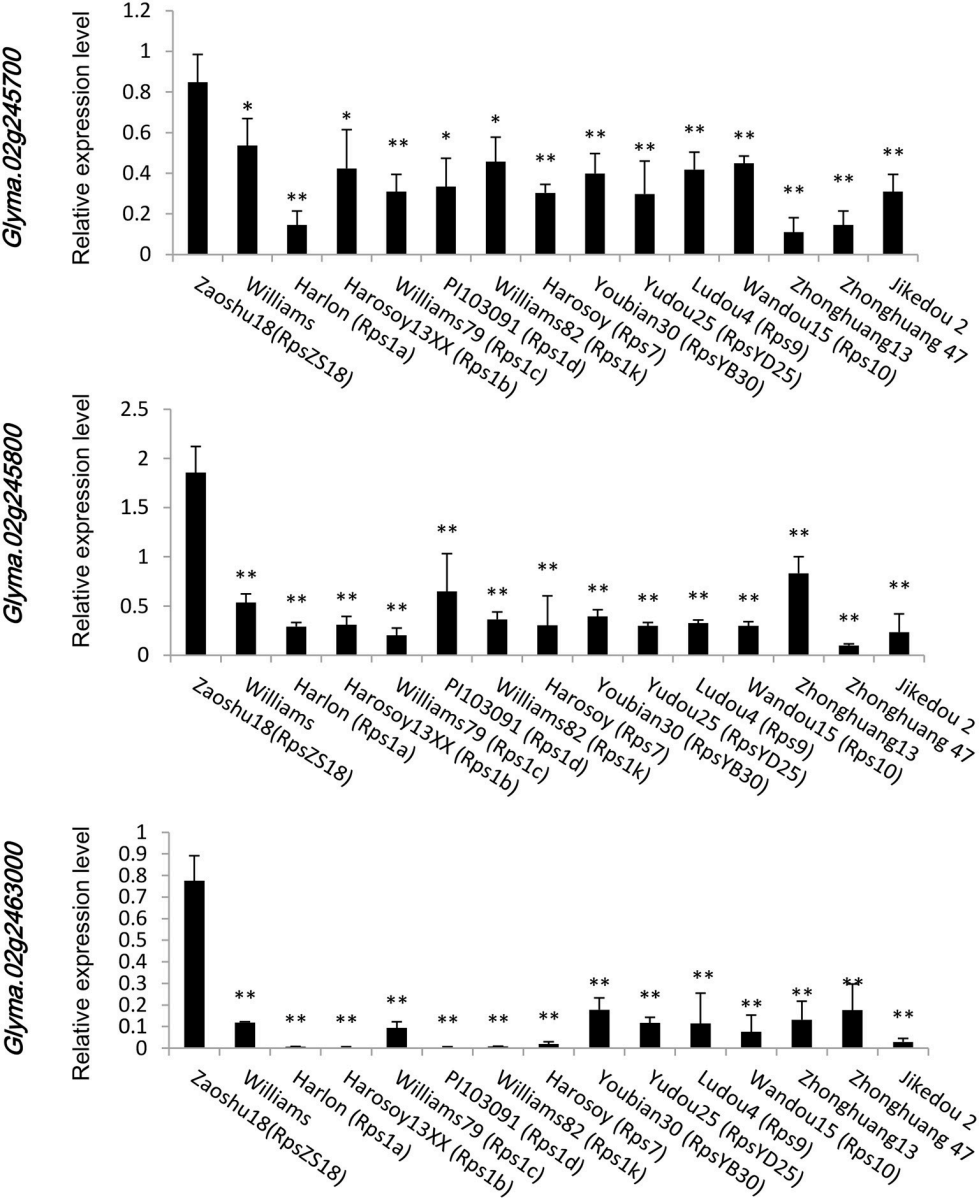
Allele expression patterns of *Glyma.02g245700, Glyma.02g245800*, and *Glyma.02g246300* in the 14 sequenced soybean cultivars and the reference genotype Williams82 at 24 h post inoculation. Three biological replicates with their respective three technical replicates were performed and statistically analyzed using Student's *t*-test (^*^P < 0.05, ^**^P < 0.01). Bars indicated standard error of the mean.

To confirm which of these genes are related to resistance to *P. sojae*, qRT-PCRs were performed to analyze the expression pattern of the six genes in Zaoshu18 and Williams. In order to detect the expression pattern of genes more effectively, we selected two pairs of primers for each gene to confirm the gene expression pattern (Table S4). However, only three of the six genes were detected; the other three genes were not detected due to low or no expression, no matter in root, stem, or leaf tissues using the two pairs of primers or redesigned primers. The three genes (*Glyma.02g245700, Glyma.02g245800, and Glyma.02g246300*) had different expression levels in different tissues and at all the time points between Zaoshu18 and Williams (Figure 4, Figure S1). The expression levels in root, stem and leaf of the three genes in Zasoshu18 were higher than that in Williams at 0 h post inoculation (Figures 4A–C). The relative expression level of *Glyma.02g245700* and *Glyma.02g245800* in stem were higher than that in root and leaf. The expression level of *Glyma.02g246300* in leaf is higher in root and stem. At all six time points, the expression levels of all three genes were higher in Zaoshu18 compared with in Williams (Figures 4D–F). In Zaoshu18, the highest expression level of *Glyma.02g245700* was at 6 h after inoculation, whereas in Williams, *Glyma.02g245700* expression was highest at 6 and 12 h and then reduced at 24, 48, and 72 h after treatment. The expression level of *Glyma.02g245800* in Zaoshu18 was up-regulated at 6, 12, 24, and 48 h after treatment. Overall, the expression trend of *Glyma.02g246300* in Zaoshu18 was down-regulated compared with at 0 h, but the expression level in Williams was so low that it was almost undetectable. These results show that these three genes had different expression levels in Zaoshu18 and Williams and were up- or down-regulated after *P. sojae* infection. Therefore, the three genes that have different expression levels between resistant and susceptible parents were preferred as resistance candidate gene. Allele expression patterns were tested for the 14 sequenced soybean cultivars and the reference genotype Williams82 at 24 h post inoculation. The expression levels of these three candidate genes in Zaoshu18 were significantly higher than those in the other 14 soybean cultivars (Figure 5).

## Discussion

Using soybean cultivars that express resistance genes is the safest and most economical way to reduce yield losses caused by PRR (Sugimoto et al., 2012). However, disease resistance provided by the expression of an *Rps* gene generally lasts for 8–15 years because of the emergence of new *P. sojae* pathotypes (Schmitthenner, 1985). Therefore, researchers continue to search for new *Rps* genes. Furthermore, mapping genes and developing closely linked markers can greatly facilitate marker-assisted pyramiding of *Rps* genes. Previous studies have demonstrated that the soybean cultivar Zaoshu18 exhibited elite resistance to PRR (Zhu et al., 2006; Yao et al., 2010), and that *RpsZS18* provided resistance against more *P. sojae* pathotypes in China than *Rps1c* and *Rps1k* (Zhang et al., 2014).

In this work, we found that the reaction of Zaoshu18 to the 14 isolates was distinct from that of the other soybean cultivars tested (Table 1). Genetic analyses of resistance against four *P. sojae* isolates, PsFJ2, PsFJ3, PsUSAR2, and Ps41-1, revealed resistance to PRR in Zaoshu18 was controlled by a single dominant gene, which validated our previous result (Yao et al., 2010). The combination of the four isolates can defeat all the identified *Rps* genes except *RpsYD29*, however, *RpsYD29* was identified on chromosome 3 (Zhang et al., 2013a) (Table 1). From the phenotypic evaluation, we concluded that Zaoshu18 contained a novel *Rps* gene. Four *P. sojae* isolates differing in virulence were used to assess a mapping population to obtain accurate PRR phenotypes. We mapped the likely location of the *RpsZS18* gene in the soybean genome to a 145.9-kb fragment between SSR markers ZCSSR33 (0.9 cM) and ZCSSR46 (0.5 cM) on chromosome 2, and created a genetic linkage map with higher resolution than the previous map of Yao et al. (2010). The order of all of the SSR markers distributed in our linkage map was consistent with the order in the Williams 82 physical map (Schmutz et al., 2010; Song et al., 2010). A SSR marker, Satt172, was found to co-segregate with *RpsZS18*.

More than 20 *Rps* genes conferring resistance to PRR have been identified on nine soybean chromosomes, but *RpsZS18* is the only *Rps* gene that has been identified on chromosome 2. On chromosome 2, three quantitative trait loci (QTL) for PRR resistance have been identified, but these QTLs are not close to the region containing *RpsZS18*, as determined in this study (Burnham et al., 2003; Han et al., 2008; Li et al., 2010). All the three QTLs are closely linked to the marker Sattt579 on chromosome 2, but the location of the marker on the reference genome Williams82 is at the position 19688108-19688299 which is far away the genomic location of *RpsZS18*. Some studies have reported several disease resistance genes to soybean mosaic virus (SMV) and soybean cyst nematode on soybean chromosome 2 (Hayes et al., 2000; Wang et al., 2004; Zhan et al., 2006; Yuan et al., 2008; Ma et al., 2017). QTLs for insect-resistance have also been detected on chromosome 2, including four QTLs effective against budworms, one QTL conferring resistance against *Megacopta cribraria*, and one QTL that offers protection from *Prodenia litura* (Rector et al., 1998; Terry et al., 2000; Xing et al., 2008). But the locations of these resistance genes or QTLs were far away from *RpsZS18*. These results indicated that *RpsZS18* is a novel disease-resistance locus.

Identification of candidate genes for disease resistance is imperative for analyzing the mechanism of resistance to pathogens in plants and will be useful in future cloning of resistance genes. However, mapped target regions generally contain many genes with different functional annotations, and a lack of SNP markers which can be used to detect gene recombination in mapping population makes it difficult to confirm *Rps* candidate genes. Generally, only genes that encode proteins with predicted disease resistance-related structures are selected as resistance genes, and there is no way to exclude other types of genes in a target region (Zhang et al., 2013b; Li Y. et al., 2017; Niu et al., 2017). Among the identified *Rps* genes, only *Rps1k* has been cloned and characterized. A NBS-LRR-encoding gene cluster was identified in the region of *Rps1k* (Gao et al., 2005; Gao and Bhattacharyya, 2008). Among all known types of R genes, more than 80% of characterized R genes are located close to NBS-LRR-encoding genes, including *Rps1k* (Shao et al., 2016). However, in this study, no typical NBS-LRR resistance gene was found in the mapped region of *RpsZS18*, which may indicate that *RpsZS18* is an *Rps* gene with a novel Phytophthora resistance mechanism. To further investigate this idea, we applied WGRS analyses. Because the four *P. sojae* isolates used to evaluated the phenotypes of the mapping population were able to defeat all the sequenced soybean cultivars containing known *Rps* genes except *RpsZS18* in this study, it suggested that the other sequenced *Rps*-containing cultivars do not contain *RpsZS18*. Hence, *RpsZS18* haplotype is absent from the other resistant varieties. Fourteen *RpsZS18* allelic haplotypes sequence of soybean genotypes were reconstructed by substituting the bases with confident variant calls in the reference genome. Identification of varients in gene region including promoter, UTR, extron or intron region is an effective way of discovering candidate genes, and this approach has been used to identify candidate genes for multiple plant diseases in recent years (Silva et al., 2012; Sudheesh et al., 2015; Singh et al., 2016a,b; Li B. et al., 2017; Li W. et al., 2017; Pandey et al., 2017). Allele haplotype sequences containing the respective SNPs and InDels are aligned with each other. The soybean cultivars that were re-sequenced did not contain *Rps* genes on chromosome 2, so, when the corresponding *RpsZS18* alleles were compared among the other sequenced soybean genotypes, genes that contained Zaoshu18-specific haplotype sequences were likely to be *Rps* candidate genes. By haplotype analyses, we identified six gene models that contained Zaoshu18-specific haplotype sequences, and all of them were in the 71.3-kb candidate genomic region of *RpsZS18*. These two results revealed that these six genes co-segregated with *RpsZS18* in the F_2:3_ population and formed a specific *RpsZS18* haplotype.

To further confirm which of these genes were expressed differently between the parental cultivars after treatment with *P. sojae*, qRT-PCR analysis was applied, and differential expression patterns of three genes were detected at different time points between Zaoshu18 and Williams. Differentially expressed genes in soybean that are responsive to *P. sojae* were found to peak at 24 h after inoculation or infection (Moy et al., 2004; Zhao et al., 2015). Further analysis of allelic genes showed that the expression levels of the three candidate genes were significantly higher than those of other sequenced soybean genotypes, indicating that these three specific haplotype genes have particular expression patterns in Zaoshu18. *Glyma.02g245700* encodes a protein with an EF-hand calcium-binding domain, which is important in the regulation of calcium signaling. Calcium is the most widely accepted messenger and plays an important role in diverse physiological stimuli, stresses, and pathogen attack, including Phytophthora (Yoshioka et al., 2003; Gao et al., 2014; Chen et al., 2015). Plants contain two major immune responses to defend against pathogen infections: pathogen-associated molecular pattern (PAMP)-triggered immunity (PTI) and effector-triggered immunity (ETI) (Jones and Dangl, 2006; Boller and He, 2009). The EF-hand domain can bind calcium directly and trigger PAMPs or effectors, which activate complex downstream responses (Lecourieux et al., 2006; Gao et al., 2014). The expression of the gene models in Zaoshu18 was significantly higher than their expression in the susceptible parent Williams, suggesting that Zaoshu18 could react more effectively through the calcium regulatory network.

*Glyma.02g245800* encodes a sugar kinase that belongs to the pfkB-type carbohydrate kinase family. pfkB-type carbohydrate kinases are involved in energy and carbohydrate metabolism (Gilkerson et al., 2012). But in a study of plant and pathogen interactions, a pfkB-type carbohydrate kinase was up-regulated in the non-host resistance reaction of *Oryza sativa* to wheat leaf rust fungus (Li et al., 2012). pfkB-type carbohydrate kinase was also up-regulated in the susceptible host–pathogen interaction between wheat and leaf rust fungus (Rampitsch et al., 2006). A proteomics study in soybean lines resistant and sensitive to *P. sojae* indicated that the percentage of differentially abundant protein involved in energy was significantly higher in resistant lines than in susceptible lines (Zhang et al., 2011). These results suggested that pfkB-type carbohydrate kinase may play an important role in the energy regulation in soybean during pathogenic infection. We found that the expression level of *Glyma.02g245800* was significantly higher in Zaoshu18 than in the susceptible cultivar Williams at all six time points, probably because expression of this gene effectively improved energy production to help the plant resist the invasion of *P. sojae*.

*Glyma.02g246300* had no annotation. BLAST searches against NCBI databases did not identify any annotated genes with high similarity to *Glyma.02g246300*. The expression level of *Glyma.02g246300* was reduced after *P. sojae* inoculation in Zaoshu18, but was almost undetectable in Williams. It may be that *Glyma.02g246300* expression was inhibited by the pathogen *P. sojae*, but lost its function in the susceptible cultivar Williams. *Glyma.02g246300* may be a novel gene, and further cloning experiment are needed to clarify its structure and function.

The other three genes were not detected by the qRT-PCRs because of their low expression levels. However, two of the three genes were annotated as related to resistance plant pathogen. *Glyma.02g2459000* is calcium-binding EF-hand protein like *Glyma.02g245700*, but the gene model only contains one 255-bp extron. *Glyma.02g246000* encodes a STK-LRR, and STK-LRRs are involved in transmembrane signal transductions and are important for plant disease resistance (Hardie, 1999; Fluhr and Kaplan-Levy, 2002). Many resistance genes in plants encode enzymes with structures similar to those of STKs, including *Xa21, Xa21D, Xa26*, and *Lr10* (Song et al., 1995; Feuillet et al., 1997; Wang et al., 1998; Sun et al., 2004). Recently, two genes encoding STK-LRRs were predicted as the *Rps* candidate genes, *RpsQ* and *RpsHN* (Li Y. et al., 2017; Niu et al., 2017). Based on these results, we consider the two genes may also contribute to the resistance of *RpsZS18*.

Although we identified three genes that were differentially expressed in resistant and susceptible species, and confirmed these candidate genes were likely to be involved in the resistance of Zaoshu18, the WGRS did not account for all the genomic information, and the annotation of the Williams82 reference genome information did not fully represent the annotation of the Zaoshu18 genome. WGRS can detect only SNPs and InDels, and cannot detect the insertion and deletion of large fragments; Different coverage of SNPs may indicate that some of the duplications have not been identified in this region (Figures S2, S3). Therefore, there may be other resistance genes in the locus that were not detected by re-sequencing. Only the screening and sequencing of BAC clones will be able to clear the genomic information in the mapping interval of *RpsZS18*.

*RpsZS18* is the only *Rps* gene on chromosome 2, so it should be relatively easy to combine it with other *Rps* genes in soybean breeding programs. Doing so will produce cultivars with more durable resistance to PRR, and perhaps produce cultivars with resistance to broad-spectrum pathogens. The Zaoshu18 soybean cultivar is an elite parent, and has been used to generate several soybean varieties (Liu et al., 2013). Related varieties may contain the same *Rps* genes as Zaoshu18 (Chen et al., 2008; Xia et al., 2011). The SSR and InDel markers that co-segregated with *RpsZS18* can be applied in molecular marker-assisted selection to breed more excellent soybean cultivars. These markers for Zaoshu18 will provide an effective tool as a better application for breeding materials.

In conclusion, we finely mapped the *RpsZS18* gene and identified six candidate genes, three of which were shown to be differently expressed between resistant and susceptible parental cultivars. The co-segregated markers may facilitate the tracking of *RpsZS18* in progenies for marker-assisted selection in soybean breeding programs. The candidate genes also provide novel fundamental details regarding the PRR resistance mechanism, and may be useful in future cloning experiments for the functional characterization of the *RpsZS18* gene.

## Author contributions

ZZ: conceived and designed the experiments; CZ, SS, LY, JD, and CD: performed the experiments; CZ: analyzed data and wrote the manuscript; ZZ: revised the paper; All authors read and approved the manuscript.

### Conflict of interest statement

The authors declare that the research was conducted in the absence of any commercial or financial relationships that could be construed as a potential conflict of interest.
