# Supplementary material for: Fine Mapping and Identification of a Novel Phytophthora Root Rot Resistance Locus RpsZS18 on Chromosome 2 in Soybean
Source: Front Plant Sci. 2018 Jan 30;9:44. doi: 10.3389/fpls.2018.00044 (PMC5797622; doi:10.3389/fpls.2018.00044)

Supplementary Figure S1 Expression patterns of *Glyma.02g245700*, *Glyma.02g245800*, and *Glyma.02g246300* in Zaoshu18 and Williams using different sets of primers

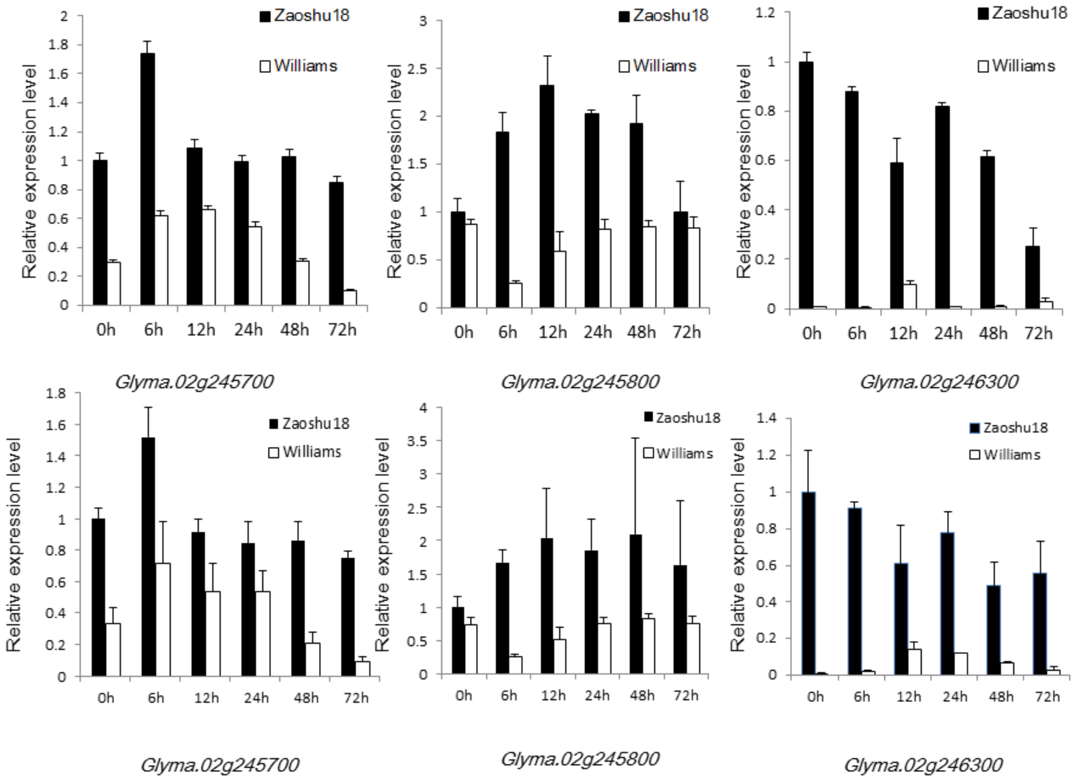

Supplement: Supplementary file 6 [file Image1.PDF]
