# Supplementary material for: Fine Mapping and Identification of a Novel Phytophthora Root Rot Resistance Locus RpsZS18 on Chromosome 2 in Soybean
Source: Front Plant Sci. 2018 Jan 30;9:44. doi: 10.3389/fpls.2018.00044 (PMC5797622; doi:10.3389/fpls.2018.00044)

**Supplement Figure S2** Read depth of called variants of the 15 sequenced soybean genotypes in the 145.9-kb mapping region.

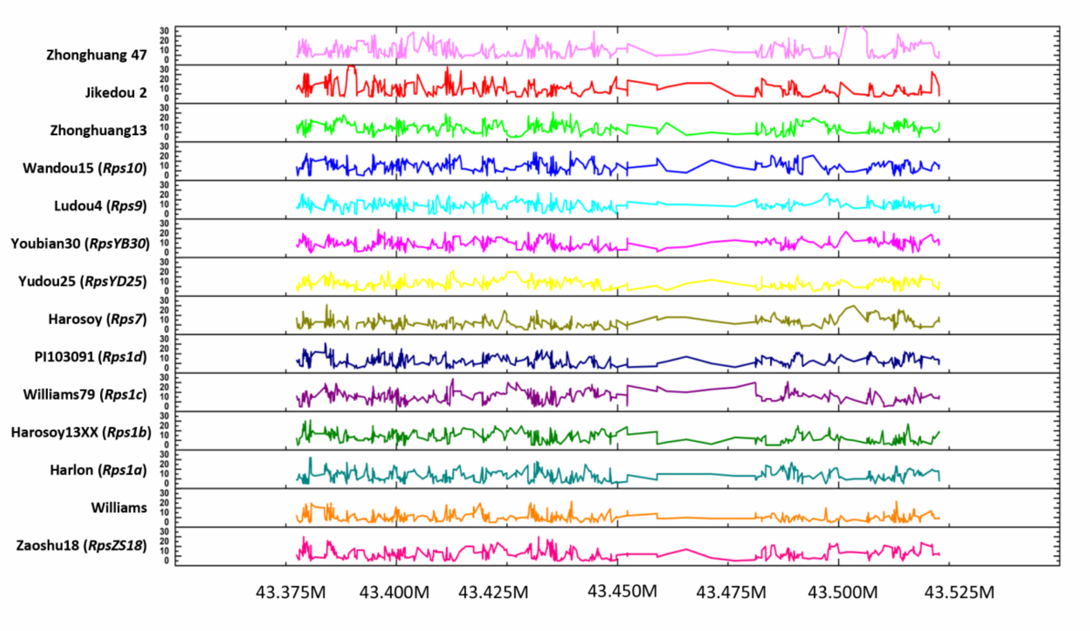

Supplement: Supplementary file 7 [file Image2.pdf]
