# Supplementary figures and images for: Fine Mapping and Identification of a Novel Phytophthora Root Rot Resistance Locus RpsZS18 on Chromosome 2 in Soybean
Source: Front Plant Sci. 2018 Jan 30;9:44. doi: 10.3389/fpls.2018.00044 (PMC5797622; doi:10.3389/fpls.2018.00044)

Glyma.03g246000 in Zaoshu18.

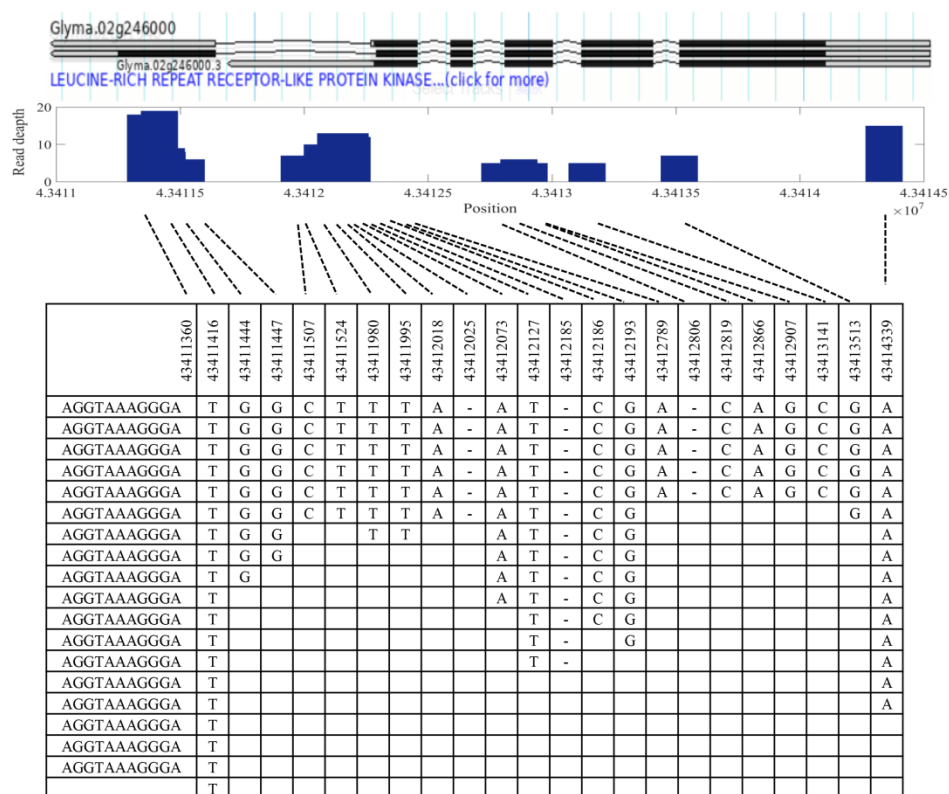

Supplement: Supplementary file 8 [file Image3.pdf]
